# Improved UV Photoresponse Performance of ZnO Nanowire Array Photodetector via Effective Pt Nanoparticle Coupling

**DOI:** 10.3390/nano14171442

**Published:** 2024-09-04

**Authors:** Nan Wang, Jianbo Li, Chong Wang, Xiaoqi Zhang, Song Ding, Zexuan Guo, Yuhan Duan, Dayong Jiang

**Affiliations:** 1 School of Engineering, Changchun Normal University, Changchun 130032, China; wangchong@ccsfu.edu.cn (C.W.); zhangxq@ccsfu.edu.cn (X.Z.); dingsong@ccsfu.edu.cn (S.D.); 2 Engineering Research Center of Jilin Province Rare Metal Deep Processing, Changchun 130022, China; 3 Engineering Research Center of Jilin Province Intelligent Manufacturing Equipment R&D and Testing, Changchun 130022, China; 4 Huadian Huijin Calcium Industry Co., Huadian 132400, China; lijianbo0325@163.com; 5 Institute for Interdisciplinary Quantum Information Technology, Jilin Engineering Normal University, Changchun 130052, China; 6 School of Materials Science and Engineering, Changchun University of Science and Technology, Changchun 130022, China; dayongjiangcust@126.com

**Keywords:** ZnO nanowire array, Pt nanoparticles, light trapping effect, surface plasmon

## Abstract

Ultraviolet (UV) photodetectors (PDs) based on nanowire (NW) hold significant promise for applications in fire detection, optical communication, and environmental monitoring. As optoelectronic devices evolve towards lower dimensionality, multifunctionality, and integrability, multicolor PDs have become a research hotspot in optics and electronic information. This study investigates the enhancement of detection capability in a light-trapping ZnO NW array through modification with Pt nanoparticles (NPs) via magnetron sputtering and hydrothermal synthesis. The optimized PD exhibits superior performance, achieving a responsivity of 12.49 A/W, detectivity of 4.07 × 10^12^ Jones, and external quantum efficiency (EQE) of 4.19 × 10^3^%, respectively. In addition, the Pt NPs/ZnO NW/ZnO PD maintains spectral selectivity in the UV region. These findings show the pivotal role of Pt NPs in enhancing photodetection performance through their strong light absorption and scattering properties. This improvement is associated with localized surface plasmon resonance induced by the Pt NPs, leading to enhanced incident light and interfacial charge separation for the specialized configurations of the nanodevice. Utilizing metal NPs for device modification represents a breakthrough that positively affects the preparation of high-performance ZnO-based UV PDs.

## 1. Introduction

UV detection technology has been widely used in many fields in recent years. Broadband semiconductor UV PDs have become a new generation of devices that have attracted much attention due to their compact size, lightweight nature, and operational independence from filters [1,2,3,4]. Among the many broadband semiconductor materials, ZnO-based materials have advantages like low defect density, high radiation resistance, and environmentally friendliness. They are considered an essential and preferred material for preparing UV PDs [5,6,7,8,9]. Many research studies have been conducted on ZnO-based PDs. However, some key issues are still unsolved, such as film defects and light absorption efficiency [10,11]. Moreover, in the future researchers should focus on preparing new ZnO-based PDs with high responsivity, low noise, high sensitivity, and low operating voltage [12,13,14].

Nanomaterials exhibit excellent optoelectronic properties due to their small size, large specific surface area, high carrier mobility, and tunable light absorption [15]. Moreover, light trapping due to the special spatial arrangement of 1D nanostructures is one of the methods that can effectively reduce light loss and has a wide range of applications in the field of room temperature photodetection [16,17,18,19]. In recent years, PDs utilizing one-dimensional (1D) NW have demonstrated notable advancements characterized by high optical conductivity gain, controllable wavelength sensitivity, rapid response times, and efficient photoelectric conversion [20,21,22,23,24]. However, unintentional doping or defects are inevitably introduced into the NW during preparation. These factors result in a high concentration of background carriers, leading to elevated dark current levels and diminished detection rates of NW PDs, thus severely constraining the devices’ detection performance [25]. Metal NPs have emerged as highly effective agents for enhancing the utilization of incident light due to their ability to generate localized surface plasmon with high spatial localization and strong local field enhancement properties [26,27]. Metal NPs are incorporated into UV PDs to enhance the radiative recombination rate and diminish the likelihood of non-radiative recombination within the devices, facilitated by the resonance effect between the matrix material and the plasma [28,29]. Leveraging the optical resonance property of plasma enhances the performance of the device across all aspects. This paper selects Pt metal NPs; the application of Pt NPs in ZnO PDs exhibits significant plasmonic effects, remarkably enhancing the responsivity of the device. There are several key reasons for choosing Pt NPs as the enhancement material instead of other metals such as Au and Ag: (1) plasmonic resonance properties, (2) chemical stability and durability, and (3) compatibility between Pt NPs and ZnO [30,31,32,33,34,35].

In this study, a high-performance UV PD was constructed by modifying a NW array of ZnO with spherical Pt NPs to address the issues mentioned earlier. The results show that the vertically structured ZnO NW array increases the interfacial area and promotes the enhancement of light trapping due to its excellent pore structure. Meanwhile, the localized surface plasmon resonance and surface depletion region induced by Pt NPs modification can significantly improve PD responsivity. These findings suggest that semiconductor surface modification utilizing Pt NPs is a practical approach to developing high-performance ZnO PDs.

## 2. Materials and Methods

**Figure 1:**
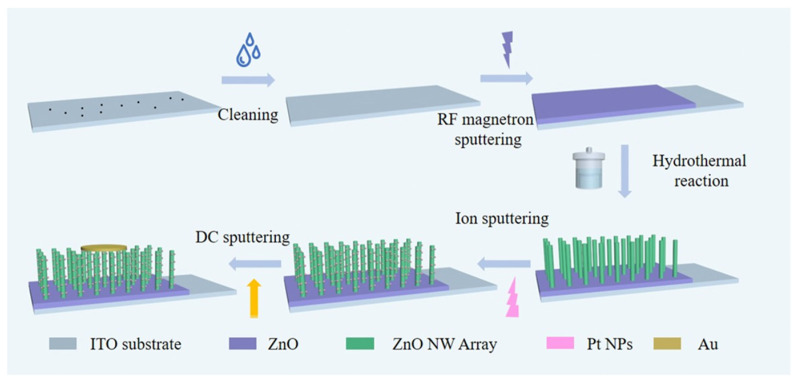
Schematic of Pt NPs/ZnO NW/ZnO PD fabrication technique.

As shown in Figure 1, the pre-grown ITO substrates were sonicated in acetone for 15 min, ethanol for 15 min, and deionized water for 10 min with the aim of removing surface contaminants, followed by blow-drying with nitrogen. Subsequently, a thin ZnO film was deposited via RF magnetron sputtering, maintaining a working pressure of 0.6 Pa, sputtering power of 150 W, and O_2_/Ar ratio of 10:40. The reaction solution was 6 mmol/L Zn(CH_3_COO)_2_-2H_2_O with 6 mmol/L HMTA, and 0.4 mL ammonia was added to promote NW growth. The substrates were reacted in an autoclave at 90 °C for 12 h to yield well-defined ZnO NW. The reacted substrate was placed in an ion-sputtering coating chamber and Pt NPs were sputtered onto ZnO NW at 30 mA for 20 s. A 20 nm thick Au layer was prepared as the top electrode using DC sputtering with high purity (99.99%) Au. For comparison, ZnO NW/ZnO PD and ZnO PD were retained.

X-ray diffraction (XRD) curves were obtained using a Rigaku X-ray powder diffractometer (Rigaku, Tokyo, Japan), utilizing Cu Kα radiation (λ = ~1.543 Å). The absorption parameters of PDs were characterized using a Lambda 950 UV/Vis/NIR spectrophotometer (Perkin Elmer, MA, USA). The morphology was characterized using a Hitachi SU8010 scanning electron microscope (SEM) (Hitachi, Tokyo, Japan). The responsivity characteristics and current-voltage of the PDs were measured at room temperature (Zolix DR800-CUST, Zolix, Beijing, China). The composition elements of PD materials were analyzed using a Hitachi SU8010 Energy Dispersive Spectrometer (EDS).

## 3. Results

**Figure 2:**
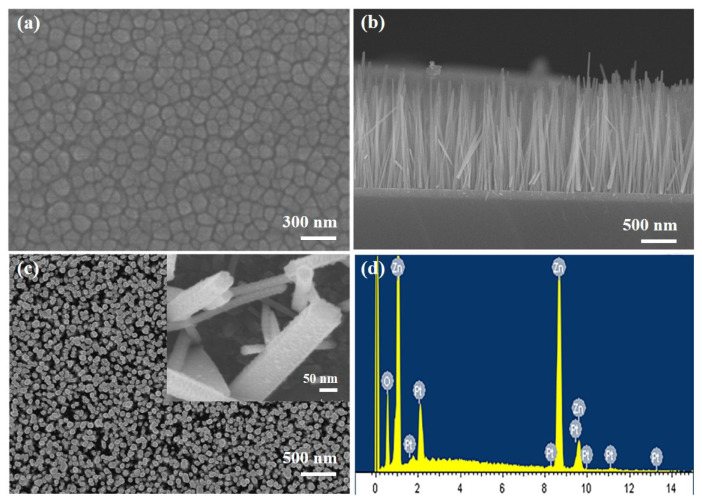
SEM Images of (**a**) top-down view of ZnO film; (**b**) cross-sections of ZnO NW/NiO films; (**c**) top-down view of ZnO NW, with insets depicting Pt NPs; and (**d**) EDS spectrum.

Figure 2a depicts a top-down view of the ZnO film, revealing a smooth surface with uniformly sized intergranular gaps of approximately 100 nm. The quality of the ZnO seed layer plays a crucial role in determining the alignment uniformity of the NW array. From the cross-section in Figure 2b, the NW grows uniformly vertical for about 1.5 μm. Figure 2c demonstrates the top-down view of the ZnO NW, where the NW grow densely and uniformly in a standard hexagonal prism shape. The inset shows a magnified image of the NW modified by Pt NPs. In Figure 2d, the EDS spectrum obtained from Pt-decorated ZnO NW confirms the presence of Pt signals.

**Figure 3:**
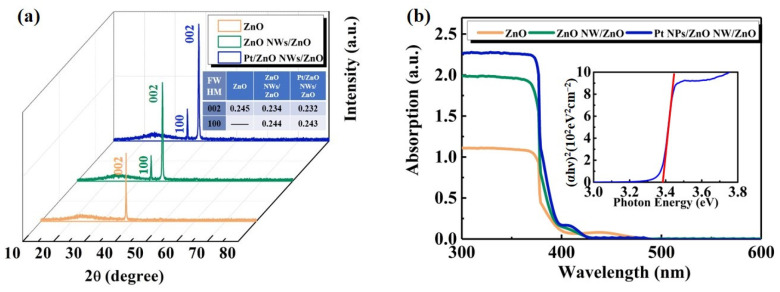
(**a**) XRD spectrum of PDs, inset table shows values of FWHM. (**b**) UV-visible absorption spectrum of PDs, inset shows (αhγ)^2^ vs. hγ.

In Figure 3a, the XRD patterns reveal diffraction peaks of ZnO at 31.5° and 34.6°, corresponding to the (100) and (002) crystal plane diffractions of ZnO vertical NW, respectively. The diffraction intensity of the ZnO NW/ZnO and Pt NPs/ZnO NW/ZnO PD shows a slight increase, indicating that the introduction of ZnO NW can compensate for the defects of thin films. During the growth of NWs, the optimization of growth conditions (such as temperature, reactant concentration, and reaction time) can be leveraged to decelerate the growth rate, allowing atoms or molecules more time to arrange in an orderly manner, thereby reducing the formation of defects [36,37]. Furthermore, due to the high specific surface area of NWs, the impact of surface energy on their growth process becomes more pronounced. To minimize surface energy, NWs tend to develop a more ordered crystalline structure with fewer defects [38]. The full width at half maximum (FWHM) is shown in the table inserted in Figure 3a. According to Scherrer’s formula, a narrower FWHM usually implies a higher crystalline quality of the crystal, a more ordered arrangement of atoms in the crystal, and no or few structural defects or impurities [39]. Therefore, ZnO NW/ZnO and Pt NPs/ZnO NW/ZnO PD have higher crystalline quality. The FWHM of the ZnO NW/ZnO PD and Pt NPs/ZnO NW/ZnO PD remains nearly identical, indicating that the sputtering deposition of Pt has no significant impact on the crystal quality of the NW. The absorption spectra of the devices were tested in the range of 300~600 nm, with (αhγ)^2^ vs. hγ plotted inside. The cutoff edge exhibits a pronounced steepness around 375 nm, suggesting that ZnO behaves as a direct bandgap semiconductor with substantial light absorption capability. The absorption coefficient exceeds 1, ensuring a robust capacity to generate photogenerated carriers. Following NW growth, the absorption intensity of the PDs notably surpasses that of the pure ZnO seed layer, accompanied by increased attenuation of UV-visible light. This enhancement can be attributed to the NW array acting like a trap, facilitating light absorption. After the sputtering of Pt NPs, the absorption of UV light by the PD shows an upward trend. The large quantities of Pt NPs lead to a stronger localized field under the excitation of UV light and effectively scatter the incident light.

**Figure 4:**
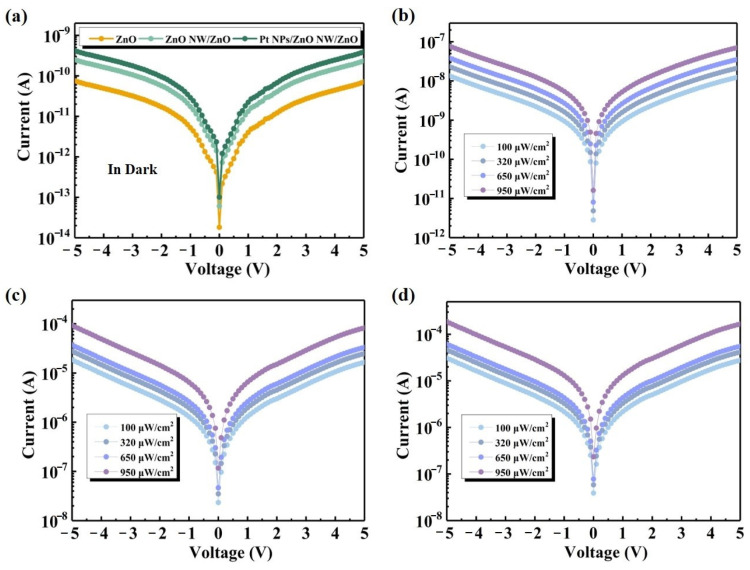
(**a**) I–V characteristics of PDs under dark conditions. I–V characteristics under UV irradiation for (**b**) ZnO PD, (**c**) ZnO NW/ZnO PD, and (**d**) Pt NPs/ZnO NW/ZnO PD.

Figure 4a displays current curves of the PDs in dark conditions. The curves for the three PD groups exhibit nonlinear characteristics centrosymmetric about the origin, suggesting a Schottky contact between the material and the fork finger electrode. The dark currents of all three sets of detectors are less than 1 nA, indicating a very low noise level in the detectors. This is primarily attributed to the high purity of ZnO material, which has fewer lattice defects and impurities. This reduction in defects and impurities minimizes the complex centers for electrons and holes, subsequently decreasing the generation of dark current. Additionally, the asymmetric electrodes contribute by optimizing the contact interface between the electrodes and the semiconductor. This optimization reduces the number of defects and traps at the interface, further decreasing the production of dark current [40]. The increased dark current observed in the ZnO NW PDs can be attributed to the higher interfacial state density of the nanostructures with larger specific surfaces. Furthermore, the sputtering of Pt NPs results in enhanced electron flow to ZnO. Consequently, the carrier concentration of the ZnO material rises. When the electrode is subjected to an applied bias, the rate of carrier directional migration increases, leading to a rise in the dark current of the device. The optical I–V characteristics of the devices at the 375 nm wavelength of the irradiated UV light and at optical power densities of 100 μW/cm^2^, 320 μW/cm^2^, 650 μW/cm^2^, and 950 μW/cm^2^ are shown in Figure 4b–d. The plot depicting the variation in photocurrent of the PDs indicates a substantial increase in the ZnO NW PDs compared to the ZnO PD, expanding by a factor of 10^3^. It indicates that more photoenergy carriers are generated on account of the spatial structure of the ZnO NW-created light trapping effect guiding the photons more efficiently. The elongated NW extends the optical path and improves the light absorption. Following the sputtering of Pt NPs, the device’s photocurrent increases under the reapplied bias, attributable to the heightened scattering efficiency of the Pt NPs and the enhanced light absorption capacity of the ZnO NW, resulting in an increased carrier concentration.

**Figure 5:**
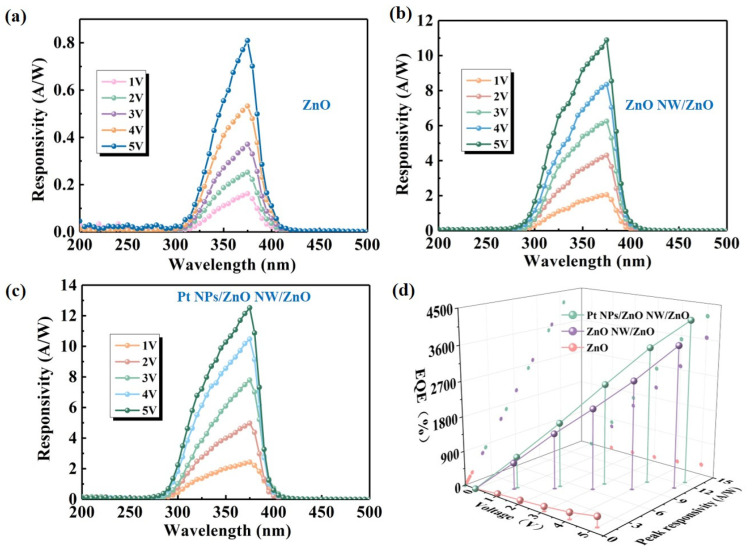
Responsivity spectra of (**a**) ZnO PD, (**b**) ZnO NW/ZnO PD, and (**c**) Pt NPs/ZnO NW/ZnO PD, along with (**d**) peak responsivity and EQE of PDs.

In order to compare the responsiveness at an optical power of 320 μW/cm^2^, the devices were tested in the range of 250 to 500 nm as in the range of absorption spectra (Figure 5a–c). The responsivity trend aligns consistently with the absorption profile trend. Responsivity is closely tied to PD absorptivity, indicating that increased incident light absorption by the materials promotes higher carrier concentration. In ZnO PDs, the responsivity varies with wavelength. Near the ZnO bandgap (approximately 375 nm), the responsivity peaks as the photon energy most closely matches the ZnO bandgap, maximizing the generation efficiency of photogenerated carriers. As the wavelength increases (and photon energy decreases), the responsivity gradually decreases because the photon energy is insufficient to excite more electrons into the conduction band. However, at shorter wavelengths (below 300 nm, for example), the responsivity may also decrease due to the saturation effect of the material absorbing high-energy photons and potential surface damage issues [41]. The responsivity of the Pt NPs/ZnO NW/ZnO PD and ZnO NW PD at 375 nm is significantly improved. The ZnO NW array with a light trapping structure has effectively increased light absorption and improved the responsivity of the device. The Pt NPs on the surface of the NW enhance the responsiveness of the device. This is attributed to the fact that Pt NPs can effectively scatter incident light under UV irradiation, and at the same time, the free electrons in Pt NPs are excited to produce collective oscillations. As a result, the local field strength in the vicinity of Pt NPs is immense, leading to enhanced incident light absorption. EQE is a vital parameter for characterizing PD photovoltaic performance and positively correlates with responsivity. The following equation describes the EQE [42]:

EQE = (R_λ_ × h × c)/(q × λ) × 100%. (1)

Here, R_λ_ is the responsivity at λ wavelength, h is Planck’s constant, c is the velocity of light, q is the charge of the electron (1.60 × 10^−19^ C), and λ is the wavelength of incident light. In order to conveniently observe the performance of the three sets of PDs, the peak responsivity and EQE data of the PDs at different bias voltages are summarized and compared in Figure 5d. The responsivity of the PD with ZnO pure film reaches just 0.81 A/W at 5 V. Following the growth of ZnO NW, the responsivity of the PD increases to about 10.89 A/W. Adding sputtered spherical Pt NPs boosts the responsivity to approximately 12.49 A/W, which is a 15-fold improvement in the optical response. Meanwhile, the EQE of the PD after sputtering Pt NPs increases from about 271% to 4190%, proving that the photodetection and photoelectric conversion efficiency of the PD is improved.

**Figure 6:**
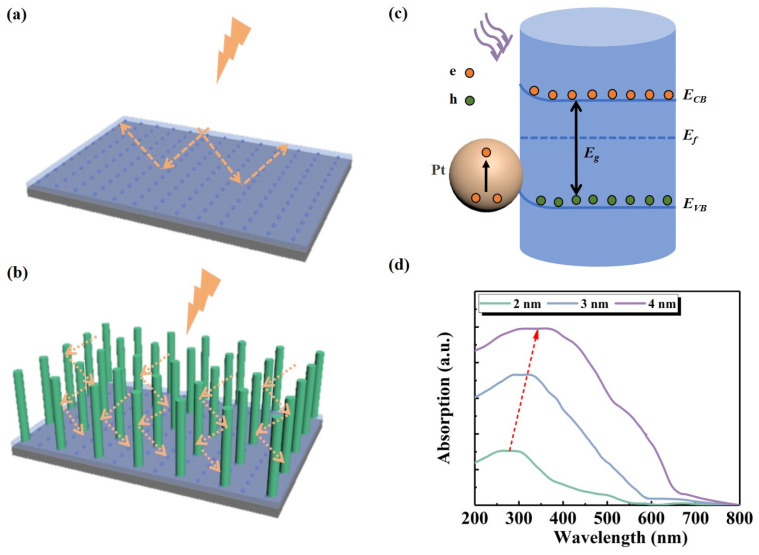
Light path diagram of PDs when illuminated (**a**) ZnO PD, and (**b**) ZnO NWs/ZnO PD. (**c**) Schematic depicting charge transfer process in Pt NPs/ZnO NW/ZnO PD under UV irradiation. (**d**) Absorption spectrum.

Figure 6a,b illustrates the schematic structure of the ZnO NW array when light is incident. When UV light irradiates the surface of a ZnO film, a substantial portion of the light is reflected without being absorbed, resulting in heavy optical losses. A high-density ZnO NW array is grown on ZnO films to construct light-trapping structures. The NW array facilitates the absorption and utilization of incident light by the PD through multiple instances of refraction, reflection, and scattering within. This is evidenced by the enhanced responsivity observed at 375 nm. The NW array acts as an effective scattering center, enabling most of the light to be coupled to the film at various angles. Light scattering improves the light absorption efficiency by increasing the photon path length and reducing the reflection of incident light [43,44,45]. The working mechanism of Pt NPs/ZnO NW/ZnO PD is explained according to the energy band theory (Figure 6c). The light-trapping effect is further intensified when Pt metals are combined with the NW-based light-trapping structure. Following the modification with Pt NPs, electrons are transferred from the conduction band of ZnO NW to the Pt NPs, owing to the larger work function coefficient of Pt (5.65 eV) than that of ZnO (3.37 eV). This process leads to the formation of depletion regions at the interface until the Fermi energy levels of Pt NPs and ZnO align. When subjected to UV irradiation, the injected photon energy generates electron-hole pairs that exceed the forbidden bandwidth of ZnO. Consequently, the formed depletion region effectively segregates the photogenerated electron-hole pairs under UV irradiation, enhancing the photocurrent [46]. At the same time, Pt NPs are directly excited via optical radiation without wavevector matching and couple the photon energy to be emitted via scattering [47]. The resonance between the metal NPs and the dielectric material in this process generates a substantial local electric field, which promotes the photogenerated carriers to be collected by the electrodes. It thus improves the quantum efficiency of the device. To validate the plasmonic effect of Pt, we conducted an additional set of experiments. Using ion sputtering technology, Pt NPs were deposited on identical quartz substrates for durations of 20 s, 40 s, and 60 s. As depicted in Figure 6d, the diameter of Pt NPs increases with longer sputtering times, accompanied by an enhancement in absorption. Notably, the absorption peak of Pt NPs is located in the broadband spectral region near 300 nm wavelength, with the absorption tail extending to cover the 375 nm wavelength region. Due to the influence of the aspect ratio, the absorption peak undergoes a redshift as the diameter of the NPs increases, demonstrating its dependence on localized surface plasmon resonance [48,49,50]. The decrease in absorption intensity of Pt NPs at longer wavelengths is attributed to the smaller coverage area of smaller particles, implying a reduced number of electrons undergoing interband transitions, thus generating lower absorption intensities [51,52]. Undoubtedly, Pt NPs contribute to the significant improvement of device performance.

**Figure 7:**
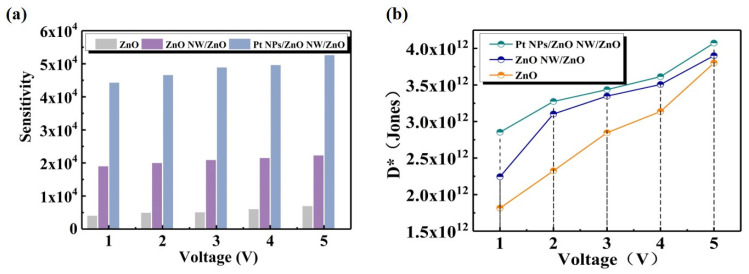
(**a**) Sensitivity and (**b**) detectivity at 1–5 V bias of PDs.

To further assess the performance of the PDs, the device sensitivity and detectivity were calculated using the responsivity and current measurements. The sensitivity of PD, defined as (*I_light_* − *I_dark_*)/*I_dark_*, is an essential quantity for the detection capability of a PD based on its current. The sensitivity of all the PDs was calculated at bias voltages from 1 to 5 V (Figure 7a). The Pt NPs/ZnO NW/ZnO PD exhibited higher sensitivity, surpassing 10^4^ at 5 V. The results demonstrate that the significant photocurrent gain induced by Pt NPs at low bias voltages is conducive to increasing the sensitivity to UV light, which dramatically improves the photodetection capability of the device. The sensitivity improvement of Pt plasma-enhanced ZnO detectors is significant. This indicates that the detector responds more sensitively to optical signals, enabling it to detect weaker light intensities. For applications requiring high sensitivity, this enhancement holds immense value. Although Pt plasmas enhance the photoelectric conversion efficiency of ZnO detectors through surface plasmon resonance, this enhancement mechanism may not be entirely linear. At higher light intensities, the gain mechanism can approach saturation, resulting in less significant increases in photocurrent compared to lower light intensities. Therefore, despite the substantial improvement in sensitivity, the increase in responsivity may be limited by this nonlinear gain mechanism [47]. The detection rate (D*) is an important parameter describing the ability of the PD to detect weak signals. The calculation formula is given below as per the reference [53]:

(2) $$D^{*}=RA^{1/2}/{(2qI_{dark})}^{1/2}.$$

In Equation (2), *R* is the responsivity, *q* is the charge of an electron, *I_dark_* is the dark current, and *A* is the light effective area (0.392 cm^2^). The D* of Pt NPs/ZnO NW/ZnO PD is increased by 2.1 times compared with ZnO PD, indicating an improved weak-signal detection capability (Figure 7b). In response to the challenge of low light absorption in ZnO PDs, devices modified with spherical Pt NPs are introduced. These modifications create a high local near-field effect in the UV band, enhancing the light-trapping structure. Consequently, it effectively improves the absorption rate of the devices for UV light and optimizes their performance.

## 4. Conclusions

In conclusion, the capability of the PD is enhanced by the dual effect of the light trapping action of the NW and the localized surface plasmon of Pt NPs. The responsivity of Pt NPs/ZnO NW/ZnO PD increases by a factor of 15 and the sensitivity by a factor of 10 compared to the ZnO PD. The introduction of spherical Pt NPs-modified devices with effective high local near-field effect in the UV band solves the bottleneck of poor detection performance of the PDs due to the low absorption light caused by short photon paths of the thin film materials, and effectively enhances the absorption of UV light by the film to improve the capabilities of the PDs. The approach proposed in this work broadens the construction of high-performance semiconductor optoelectronic nanodevices, which has potential applications in the emerging field of optoelectronics.

## Data Availability

The data presented in this study are available on request from the corresponding authors. The data are not publicly available due to their large dimensions.

## References

[1] Kaur D., Kumar M. (2021). A strategic review on gallium oxide based deep-ultraviolet photodetectors: Recent progress and future prospects. Adv. Opt. Mater..

[2] He J., Xu P., Zhou R. (2022). Combustion synthesized electrospun InZnO nanowires for ultraviolet photodetectors. Adv. Electron. Mater..

[3] Song W., Liang Z., Guo Y. (2022). 2D Ti_3_C_2_ nanoflakes anchored ZnO photodetector with 17 substantially improved deep-ultraviolet photoresponse and on/off ratio. J. Alloys Compd..

[4] Cai Q., You H., Guo H. (2021). Progress on AlGaN-based solar-blind ultraviolet photodetectors and focal plane arrays. Light Sci. Appl..

[5] Wang J.L., Chen C.H., Jin M.K. (2023). The carbon dots modified ZnO films photodetector with broadband and fast photoresponse. Opt. Mater..

[6] Kumar K.A., Mele P., Murahari P. (2022). Enhancement of performance of Ga incorporated ZnO UV photodetectors prepared by simplified two step chemical solution process. Sens. Actuators A Phys..

[7] Su L., Ouyang W., Fang X. (2021). Facile fabrication of heterostructure with p-BiOCl nanoflakes and n-ZnO thin film for UV photodetectors. J. Semicond..

[8] Li Q., Huang J., Meng J. (2022). Enhanced performance of a self-powered ZnO photodetector by coupling LSPR-inspired pyro-phototronic effect and piezo-phototronic effect. Adv. Opt. Mater..

[9] Xie H., Kang C., Iqbal M.A. (2022). Ferroelectric Tuning of ZnO Ultraviolet Photodetectors. Nanomaterials.

[10] Mondal S., Ghosh S., Basak D. (2021). Extraordinarily high ultraviolet photodetection by defect tuned phosphorus doped ZnO thin film on flexible substrate. Mater. Res. Bull..

[11] Kim D., Leem J.-Y. (2021). Crystallization of ZnO thin films via thermal dissipation annealing method for high-performance UV photodetector with ultrahigh response speed. Sci. Rep..

[12] Hui Y., Ren Y., Song J. (2022). Preparation of flexible ultraviolet photodetectors based on ZnO film using a dip-coating method on different flexible substrates. J. Phys. D Appl. Phys..

[13] Podder S., Basumatary B., Gogoi D. (2021). Pyro-phototronic application in the Au/ZnO interface for the fabrication of a highly responsive ultrafast UV photodetector. Appl. Surf. Sci..

[14] Wu C., Luo X., Yu X. (2023). Improving performance of ZnO Schottky photodetector by inserting MXenes modified-layer. Chin. Chem. Lett..

[15] Wang N., Jiang D.Y., Zhao M. (2019). Enlarged responsivity-ZnO honeycomb nanomaterials UV photodetectors with light trapping effect. Nanotechnology.

[16] Wong T.X., Wang J.J., Li J. (2021). Enhancing the device performance of SiNP array/PtTe_2_ heterojunction photodetector by the light trapping effect. Sens. Actuators A Phys..

[17] Mao J., Zhang B.C., Shi Y.H. (2022). Conformal MoS_2_/silicon nanowire array heterojunction with 19 enhanced light trapping and effective interface passivation for ultraweak infrared light detection. Adv. Funct. Mater..

[18] Shan C.C., Zhao M., Jiang D.Y. (2019). Improved responsivity performance of ZnO film ultraviolet photodetectors by vertical arrays ZnO nanowires with light trapping effect. Nanotechnology.

[19] Oh H., Kim H.J., Kim S. (2021). Highly flexible and stable perovskite/microbead hybrid photodetectors with improved interfacial light trapping. Appl. Surf. Sci..

[20] Ji Y., Wu L., Liu Y. (2021). Chemo-phototronic effect induced electricity for enhanced self-powered photodetector system based on ZnO nanowires. Nano Energy.

[21] Huang R., Lin D.H., Liu J.Y. (2021). Nanochannel-confined growth of crystallographically orientated perovskite nanowire arrays for polarization-sensitive photodetector application. Sci. China Mater..

[22] Wang N., Gu Z.W., Guo Z.X. (2023). Improved high-performance ultraviolet photodetectors through controlled growth of ZnO lateral nanowires. J. Lumin..

[23] Li Z.Y., Trendafilov S., Zhang F.L. (2021). Broadband GaAsSb nanowire array photodetectors for filter-free multispectral imaging. Nano Lett..

[24] Noh Y., Jeong H., Lee D. (2021). Enhanced ultraviolet photodetector using zinc oxide nanowires with 20 intense pulsed light post-treatment. J. Alloys Compd..

[25] Yalagala B.P., Dahiya A.-S., Dahiya R. (2023). ZnO nanowires based degradable high-performance photodetectors for eco-friendly green electronics. Opto-Electron. Adv..

[26] Noh Y., Shin J., Lee H. (2021). Decoration of Ag nanoparticle on ZnO nanowire by intense pulsed light and enhanced UV photodetector. Chemosensors.

[27] Yu J., Lou J., Wang Z. (2021). Surface modification of β-Ga_2_O_3_ layer using pt nanoparticles for improved deep UV photodetector performance. J. Alloys Compd..

[28] Cao F., Jin L., Wu Y. (2021). High-performance, self-powered UV photodetector based on Au nanoparticles decorated ZnO/CuI heterostructure. J. Alloys Compd..

[29] Sun X., Sun J., Xu J. (2021). A Plasmon-Enhanced SnSe_2_ Photodetector by Non-Contact Ag Nanoparticles. Small.

[30] Guo Z., Jiang D., Zhao M. (2016). Surface plasmon enhanced the responsivity of the ZnO/Pt nanoparticles/ZnO sandwich structured photodetector via optimizing the thickness of the top ZnO layer. Solid-State Electron..

[31] Aigner W., Bienek O., Desta D. (2017). Optoelectronic properties and depth profile of charge transport in nanocrystal films. Phys. Rev. B.

[32] Periyanagounder D., Wei T.C., Li T.Y. (2020). Fast-response, highly air-stable, and water-resistant organic photodetectors based on a single-crystal Pt complex. Adv. Mater..

[33] Agrawal J., Dixit T., Palani I.A. (2019). Development of reliable and high responsivity ZnO-based UV-C photodetector. IEEE J. Quantum Electron..

[34] Guo Z., Zhao M., Jiang D. (2021). A sandwich-structured surface plasmon ultraviolet photodetector based on ZnO thin film. J. Mater. Sci. Mater. Electron..

[35] Yu M., Wan P., Tang K. (2024). Plasmonically-boosted high-performance UV self-biased photodetector based on SiC-based low-dimensional heterojunction via Pt nanostructures deposition. Surf. Interfaces.

[36] Wang F., Wang C., Wang Y. (2016). Diameter dependence of planar defects in InP nanowires. Sci. Rep..

[37] Liu X., Pandey A., Mi Z. (2021). Nanoscale and quantum engineering of III-nitride heterostructures for high efficiency UV-C and far UV-C optoelectronics. Jpn. J. Appl. Phys..

[38] Si P., Zheng Z., Gu Y. (2023). Nanostructured TiO2 arrays for energy storage. Materials.

[39] Rani N., Ahlawat R. (2018). Tailoring the structural and optical parameters of Eu^3+^: CeO^2^-SiO_2_ nanopowder via thermal treatment. Silicon.

[40] Hwang J.D., Chang W.T., Chen Y.H. (2007). Suppressing the dark current of metal–semiconductor–metal SiGe/Si heterojunction photodetector by using asymmetric structure. Thin Solid Film..

[41] Hu J., Chen J., Ma T. (2023). Research advances in ZnO nanomaterials-based UV photode tectors. Nanotechnology.

[42] Xiao L., Chen S., Chen X. (2018). High-detectivity panchromatic photodetectors to the near infrared region based on a dimeric porphyrin small molecule. J. Mater. Chem. C.

[43] Wang N., Jiang D.Y. (2021). Light trapping in ZnO nanowires to control ultraviolet photodetection responsivity. J. Mater. Sci..

[44] Ning L., Jiang T.H., Shao Z.-B. (2018). Light-trapping enhanced ZnO-MoS2 core–shell nanopillar arrays for broadband ultraviolet-visible-near infrared photodetection. J. Mater. Chem. C.

[45] Garnett E., Yang P. (2010). Light trapping in silicon nanowire solar cells. Nano Lett..

[46] Zhang X., Liu Q., Liu B. (2017). Giant UV photoresponse of a GaN nanowire photodetector through effective Pt nanoparticle coupling. J. Mater. Chem. C.

[47] Pei J.N., Jiang D.Y., Zhao M. (2016). Controlled enhancement range of the responsivity in ZnO ultraviolet photodetectors by Pt nanoparticles. Appl. Surf. Sci..

[48] Langhammer C., Yuan Z., Zoric I., Kasemo B. (2006). Plasmonic Properties of Supported Pt and Pd Nanostructures. Nano Lett..

[49] Choi H.J., Kim S., Chu E.K. (2019). Enhanced photon emission efficiency using surface plasmon effect of Pt nanoparticles in ultra-violet emitter. Micromachines.

[50] Bigall N.C., Ha T., Klose M., Simon P., Eng L.M., Eychmuller A. (2008). Monodisperse Platinum Nanospheres with Adjustable Diameters from 10 to 100 nm: Synthesis and Distinct Optical Properties. Nano Lett..

[51] Rivadulla J.F., Vergara M.C., Blanco M.C., Lopez-Quintela M.A., Rivas J. (1997). Optical Properties of Platinum Particles Synthesized in Microemulsions. J. Phys. Chem. B.

[52] Luther J.M., Jain P.K., Ewers T., Alivisatos A.P. (2011). Localized surface plasmon resonances arising from free carriers in doped quantum dots. Nat. Mater..

[53] Wang P., Liu S., Luo W. (2017). Arrayed van der waals broadband detectors for dual-band detection. Adv. Mater..

